# Post-polypectomy surveillance: follow-up recommendations from the Alberta Colorectal Cancer Screening Program

**DOI:** 10.1093/jcag/gwae007

**Published:** 2024-03-19

**Authors:** Daniel C Sadowski, Michael R Kolber, Anthony Gomes, Linda Hickle, Robert Hilsden, David Ross McLean, Dereck Mok, Barbara Moysey, Nicole Nemecek, John David Ryan, Richard Sultanian, Jessica Wiseman, Huiming Yang

**Affiliations:** Division of Gastroenterology, University of Alberta, Edmonton, Canada, T6G 2X8; Department of Family Medicine, University of Alberta, Canada, T6G 2T4; Coaldale Colorectal Screening Program, Lethbridge, AB, Canada, T1M 1L2; SCOPE Program, Alberta Health Services, Canada, T5J 0K1; Department of Community Health Sciences and Department of Medicine, Cumming School of Medicine, University of Calgary, Calgary, AB, Canada, T2N 4N1; Department of Pathology, Royal Alexandra Hospital, Edmonton, Canada, T5H 3V9; Division of General Surgery, University of Alberta, Edmonton, Canada, T6G 2B7; Alberta Colorectal Cancer Screening Program, Alberta Health Services, Canada, T2S 3C3; Alberta Colorectal Cancer Screening Program, Alberta Health Services, Canada, T2S 3C3; Central Alberta Digestive Disease Specialists, Red Deer, Alberta, Canada, T4N 1C7; Division of Gastroenterology, University of Alberta, Edmonton, Canada, T6G 2X8; Alberta Colorectal Cancer Screening Program, Alberta Health Services, Canada, T2S 3C3; Alberta Colorectal Cancer Screening Program, Alberta Health Services, Canada, T2S 3C3

**Keywords:** colonoscopy, polypectomy, surveillance

## Abstract

In 2013, the Alberta Colorectal Cancer Screening Program (ACRCSP) initially published recommendations for post-colonoscopy follow-up and polypectomy. Over time, emerging evidence and evolving surveillance guidelines from various expert groups necessitated a comprehensive review to align with the healthcare landscape in Alberta. To accomplish this, an expert panel was convened. Using the Agree II tool, we identified high-quality Clinical Practice Guidelines that were relevant to the Alberta medical context. Recommendations from these guidelines were adapted to fit the specific needs of Alberta. Recognizing inconsistencies and gaps within the existing guidelines, we conducted targeted literature reviews to ensure a comprehensive and evidence-based approach to our recommendations.

Our revised recommendations build upon the assumption that a high-quality index colonoscopy has been performed at baseline. They are intended to enhance the quality of care and reduce unnecessary procedures. As well, they align with the growing consensus in the scientific literature that individuals with low-risk tubular adenomas may not require aggressive colonoscopy surveillance.

The updated Alberta recommendations aim to provide clear recommendations for practicing endoscopists, referring physicians, and their patients. They address crucial questions such as determining which patients should commence surveillance via colonoscopy and which individuals should return to average-risk screening using the fecal immunochemical test (FIT). Additionally, our recommendations outline the appropriate surveillance intervals for those requiring continued monitoring.

## Graphical Abstract

**Graphical Abstract:**
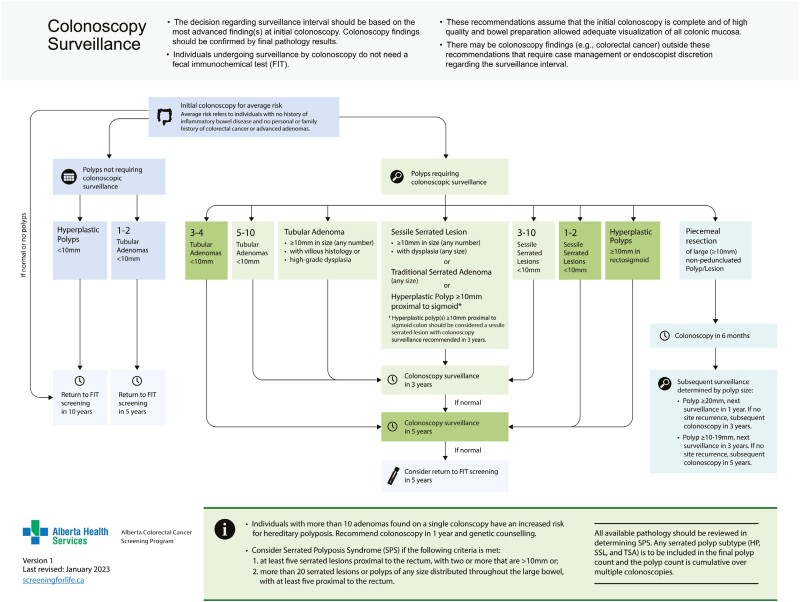
ACRCSP Recommendations for Post-Polypectomy Surveillance Summary

## Introduction

Established in 2007, the Alberta Colorectal Cancer Screening Program (ACRCSP) is a comprehensive provincial initiative led by Alberta Health Services (AHS). It operates in collaboration with healthcare providers to conduct organized screening for colorectal cancer. The program involves regular screening for individuals at average risk, typically every 1-2 years starting at age 50, using the fecal immunochemical test (FIT). Additionally, for individuals who have a first-degree relative diagnosed at age less than 60 with colorectal cancer, colonoscopy is recommended.

Annually, more than 100,000 colonoscopies are performed by a range of medical professionals, including gastroenterologists, general surgeons, and family physicians, across over 50 endoscopy units in Alberta. Notably, over 30% of these colonoscopies are conducted for surveillance purposes.

The ACRCSP initially issued recommendations for post-polypectomy surveillance in 2013. Recently, due to emerging evidence and evolving guidelines, there has been a need to revise and update these recommendations, particularly regarding the surveillance of low-risk adenomas and polyps. The purpose of this effort is to provide current and evidence-based recommendations for monitoring patients who have undergone colonic polypectomy.

## Methodology

Our goal was to offer practitioners a set of recommendations tailored to the Alberta context, based on the latest Clinical Practice Guidelines (CPGs). We used the AGREE II appraisal tool to select high-quality existing CPGs.^1^ Two evaluators with expertise in critical appraisal, working within AHS Screening Programs, independently assessed the CPGs. Any significant discrepancies in their scoring (>2-point difference) were resolved by each evaluator revisiting the guideline independently and rescoring. This was not a formal Clinical Practice Guideline process as we primarily relied on the systematic literature reviews already included in existing guidelines. However, we did perform selective supplementary literature reviews in cases where new literature became available or when an existing CPG did not address a topic relevant to the Alberta context.

Recommendations for post-polypectomy follow-up were created for each distinct polyp type rather than using confusing terminology such as low or high-risk adenomas.

The following guiding principles were adhered to in formulating evidence reviews and recommendations:

- Improve population health. The goal of screening is to reduce colorectal cancer mortality and incidence. Surrogate markers such as the occurrence of advanced adenomas were given less weight in the decision making.^2^
- Reduction of harms. Colonoscopy is an invasive procedure not without risk. The benefits of surveillance colonoscopy need to be maximized while the potential harms are minimized. As well, a potential increase in cancer incidence due to decreased surveillance needs to be considered.
- Costs and resource allocation. High demand for colonoscopy resources requires strategic alignment with those patients most likely to benefit from the procedure.

The ACRCSP appointed 2 co-chairs for this initiative (DCS and MRL). The co-chairs identified and appointed stakeholders for the formation of a 10-member panel, including gastroenterologists (4), surgeons (2), and a family medicine practitioner who performs endoscopy (1). The panel also included 2 specialists nurses and 1 pathologist. Panel members were selected for their expertise in systematic review or because of their leadership roles in colonoscopy and colon cancer screening provincially or nationally. The expert panel met in a series of 9 virtual meetings held over 10 months. After review of the relevant evidence, proposed wording of each recommendation was discussed and iterated with feedback. An anonymous online voting tool was used to assess agreement with a given recommendation. As part of the decision-making process, an existing 2013 recommendation would remain unchanged unless there was new/emerging evidence since the previous update or if consensus (50% of members plus 1) could not be achieved.

## Results

The total AGREE II scores were calculated and are presented in Supplementary Appendix 1. The overall scores ranged from 58% (Ontario) to 92% (United Kingdom, Australia).^3,4^ Significant discrepancies in scoring occurred 4 times (<4% of items) and were resolved by appraisers revisiting the guideline and rescoring. However, the expert panel found that the 2 guidelines with the highest scores (United Kingdom, AUS) contained recommendation wording that was imprecise and difficult to apply to the Alberta milieu. For example, many of the UK recommendations assumed the existence of a populated-based screening program, a situation that does not apply to Alberta. As well, the panel identified that the USMSTF and CCO guidelines historically have been very influential in Alberta. Based on this, the panel selected three clinical practice guidelines (ESGE 2020, USMSTF 2020, and CCO 2019)^5–7^ most relevant to clinical practice in Alberta. Similarities or differences in key recommendations of preferred guidelines are summarized in Supplementary Appendix 2.

Members were asked to agree or disagree by voting on 14 proposed statements. Unanimity (100%) was achieved for 12 of the 14 recommendations. Tabulation of panel consensus for each statement is found in Supplementary Appendix 3.

## Recommendations for post-polypectomy surveillance

The panel recommends that all clinicians consider the following caveats in using the new recommendations in advising the appropriate follow-up interval for patients who have had colonic polyps removed:

- Ensure that a high-quality baseline colonoscopy has been performed. A high-quality colonoscopy is one where: the cecum is reached with photo documentation, bowel preparation allows adequate visualization of all colonic mucosa, with a recommended minimum withdrawal time, with complete removal of all polyps seen, and with documentation that meets endoscopy reporting standards.^8^
- Polyp size is objectively estimated in reference to either snare diameter or open biopsy forceps.
- All polypectomies are carried out with good technique and all polypectomy material is sent to pathology.^9^
- The colonoscopy procedure report should clearly state who is responsible for arranging the follow-up colonoscopy.
- The decision regarding surveillance interval should be based on the most advanced finding(s) at the initial colonoscopy. Colonoscopy findings should be confirmed by final pathology results.
- Surveillance recommendations also need to consider baseline risk for CRC based on other factors such as family history (outside the scope of this work).^10^

*Initial colonoscopy finding of*: **Normal or no polyps**

**RECOMMENDATION:**

**For an average risk patient with no polyps or normal findings on colonoscopy, the panel recommends FIT in 10 years.**

Evidence confirms that average-risk individuals who have a normal initial colonoscopy have a decreased future risk of colorectal cancer to below that of unscreened populations.^11^ Average risk refers to individuals who are asymptomatic, no history of inflammatory bowel disease and no personal or family history of colorectal cancer or advanced adenomas. Currently, the ACRCSP recommends average-risk screening with FIT every 1–2 years for those aged 50–74. The current FIT threshold in Alberta is 75 ng Hb/mL. The panel recommends that average risk patients should rescreen with FIT in 10 years, following a normal initial colonoscopy with no polyps.

These surveillance recommendations also need to consider baseline risk for colorectal cancer (CRC) based on family history or other heritable factors or existing illness (such as inflammatory bowel disease (IBD)) and adjustments may need to be made within the 10-year interval.

Voting results:

Unanimous decision (10/10).

*Initial colonoscopy finding of*: hyperplastic polyp(s) <10 mm

**RECOMMENDATION:**

**For an average risk patient with finding(s) of hyperplastic polyp(s) <10 mm, the panel recommends FIT in 10 years.**

Hyperplastic polyps (HP) in the rectosigmoid are common findings at colonoscopy and can be readily identified by their typical appearance using image enhancement such as electronic chromoendoscopy. Small rectal HP’s do not routinely require endoscopic removal.

The presence of up to 20 HPs has not been associated with increased subsequent risk of CRC.^12^ However, the finding of more than 20 HPs, especially if found proximal to sigmoid colon should lead to consideration of serrated polyposis syndrome that carries an increased risk of subsequent CRC.

The expert panel recommended FIT in 10 years. In Alberta, FIT is the entry-level CRC screening test for average risk populations.

Voting results:

Unanimous decision (10/10).

*Initial colonoscopy finding of*: hyperplastic polyp(s) ≥10 mm.

**RECOMMENDATION:**

**For a colonoscopy finding of hyperplastic polyp(s) ≥10mm:**

1. **Proximal to sigmoid colon, the panel recommends colonoscopy in 3 years.**
2. **In rectosigmoid, the panel recommends colonoscopy in 5 years.**

Review of currently existing CPGs: (Supplementary Appendix 2):

- European Society of Gastrointestinal Endoscopy (ESGE) concluded that serrated polyps ≥10 mm and serrated polyps with dysplasia yield similar metachronous advanced neoplasia or CRC and recommended surveillance at 3 years.^5^
- United States Multi-Society Task Force (USMSTF) recommends colonoscopy in 3–5 years for HP ≥10 mm. A 3-year follow-up is favored if concerns about consistency in distinguishing SSP from HP locally.^6^

There is large inter-observer variability in pathologists when distinguishing between SSL’s and HP’s. Histologically, it may be difficult to distinguish between SSL’s and HP’s particularly if the specimen sectioning is not optimal to see entire crypts in the specimen. As a result, proximal colonic serrated lesions >10 mm in size that are deemed to be HP may be considered SSL’s by clinicians. Colonoscopy at 3 years is recommended (see SSL recommendation below).

Hyperplastic polyps (HP) in the rectosigmoid are common findings at colonoscopy and can be readily identified by their typical appearance using image enhancement such as electronic chromoendoscopy. Small rectosigmoid HP’s (<10 mm) do not routinely require endoscopic removal. Larger rectosigmoid lesions should be removed and if hyperplastic, colonoscopy in 5 years is recommended.

The presence of up to 20 HPs has not been associated with increased subsequent risk of CRC.^12^ However, the finding of more than 20 HPs, especially if found proximal to sigmoid colon should lead to consideration of serrated polyposis syndrome that carries an increased risk of subsequent CRC.

Voting results:

Unanimous decision (10/10).

*Initial colonoscopy finding of*: 1 or 2 tubular adenoma(s) <10 mm.

**RECOMMENDATION:**

**For a colonoscopy finding of 1 or 2 tubular adenoma(s) <10 mm, the panel recommends FIT in 5 years.**

Recent studies suggest that patients who undergo colonoscopy with removal of adenomas less than 10 mm without evidence for high grade dysplasia have a *similar* risk of CRC cancer incidence and mortality compared to patients with a normal colonoscopy and a *lower* risk of CRC compared to an age-matched unscreened population.^11–15^

Review of currently existing CPGs: (Supplementary Appendix 2):

- ESGE recommends patients with complete removal of 1–2 (<10 mm) adenomas do not require endoscopic surveillance and should be returned to screening.^5^
- USMSTF recommends colonoscopy in 7–10 years for 1–2 small adenomas (essentially average risk screening).^6^
- Within Canada, Cancer Care Ontario (CCO) recommends that low-risk adenomas (defined as 1–2 tubular adenomas [<10 mm] without high-grade dysplasia) be screened with FIT five years after their initial colonoscopy.^7^

Because of this disparity, an evidence review regarding the influence of the number of small (<10 mm) adenomas on the downstream risk for the development of colorectal cancer was performed. See Supplementary Appendices 4 and 6 for further details regarding the literature review methodology.

Based on 2 studies^13,16^ (230 000 patients with median follow up of 7–13 years) we found that patients with 1–2 TA’s compared to those with no adenomas have a slightly increased risk of CRC [RR: 1.31 (95% CI 1.05, 1.63)]. See Supplementary Appendix 6 Analysis 2. However, the included studies did not control for polyp size or pathology. Therefore, given that our clinical scenario of 1–2 adenomas <10 mm in size was not explicitly measured, the expert consensus of the panel was that this risk estimate of subsequent cancer is probably an overestimation.

This is supported by a recent meta-analysis published after our review that found a marginally elevated risk of CRC incidence and no increased risk for CRC mortality.^17^

Therefore, it is reasonable, through shared decision-making, to propose a return to FIT screening rather than colonoscopy. Since this group likely has an average risk for CRC, some guideline panels have recommended a return to average risk screening. However, due to the potential for a slightly elevated risk for increased colorectal cancer incidence, a 5-year follow-up with FIT was recommended. Additionally, from a primary care perspective, implementing a change to average risk screening in the short term was deemed too drastic. Consequently, a 5-year follow-up with FIT is suggested. This also aligns with the CCO recommendations.

For patients who are now due for their 5-year colonoscopy follow-up for 1–2 small tubular adenomas, screening with FIT is a valid option that some patients may prefer. The decision to resume surveillance with FIT or colonoscopy should be a shared decision. Some screening centers and endoscopists may not accept referrals for colonoscopy for low-risk lesions based on this new evidence.

Voting results:

Unanimous decision (10/10).

*Initial colonoscopy finding of:* 3 or 4 tubular adenomas <10 mm.

**RECOMMENDATION:**

**For a colonoscopy finding of 3 or 4 tubular adenomas <10 mm, the panel recommends colonoscopy in 5 years.**

Previous recommendations for aggressive surveillance in patients with 3 or more small adenomas were based on studies prior to 2000. Since that time, high-definition endoscopes, better bowel prep, and attention to adenoma detection rates have resulted in proportionally more small adenomas being found during endoscopy.^18^ This has resulted in a screening paradox where aggressive surveillance is recommended for lesions that may not confer an increased risk for CRC. At least 3 more recent large observational cohort studies have demonstrated that the number of non-advanced adenomas less than 10 mm in diameter does not have an impact on the risk for colorectal cancer incidence or mortality. This effect appears to extend up to and probably beyond 5 colonic adenomas.^16,18,19^

Review of currently existing CPGs (Supplementary Appendix 2):

- ESGE recommends patients with complete removal of 1–4 (<10 mm) adenomas do not require endoscopic surveillance and should be returned to screening.^5^
- USMSTF recommends colonoscopy in 3–5 years for 3–4 small adenomas.^6^
- Within Canada, CCO recommends that 3–4 tubular adenomas (<10 mm) repeat colonoscopy in 3 years.^7^

Given this disparity, an evidence review regarding the influence of the number of adenomas on the downstream risk for the development of colorectal cancer was performed. See Supplementary Appendices 4 and 6 for further details regarding the literature review methodology.

When comparing ≥ 3 adenomas to < 3 adenomas (*Analysis 1*), 5 studies involving 70 000 patients did not identify an increased risk in subsequent CRC (RR: 0.94; 0.59, and 1.49). There was considerable heterogeneity between the studies which may be the result of variable lengths of follow-up and other related factors. When comparing patients with ≥3 adenomas to patients with 0 adenomas, (*Analysis 3*) there does appear to be an increased risk of subsequent CRC: RR: 2.21 (95% CI; 1.06–4.62). While the panel noted wide CIs and considerable uncertainty in this estimate, it was felt that the ESGE’s recommendation to return to average risk screening (e.g., FIT) could not be supported by this evidence. As well, the 2013 Alberta recommendation for a 3-year follow-up colonoscopy seemed too aggressive given the low relative risk in this patient population. Thus, the panel recommended a repeat colonoscopy in 5 years. If the follow-up 5-year colonoscopy is normal or shows only 1 or 2 small TA with no high-grade dysplasia (HGD), then the interval for the subsequent examination should be 5–10 years.

Voting results:

Unanimous decision (10/10).

*Initial colonoscopy finding of:* 5–10 tubular adenomas <10 mm, or any adenoma ≥10 mm, or with villous/tubulovillous features or high-grade dysplasia.

**RECOMMENDATION:**

**For a colonoscopy finding of 5–10 tubular adenomas <10 mm, or any adenoma ≥10 mm, or with villous/tubulovillous features or high-grade dysplasia, the panel recommends colonoscopy in 3 years.**

Multiple studies have confirmed that identification of 1 or more adenomas >10 mm in size is an independent risk factor for the development of CRC.^20–22^

The ESGE has recommended that villous histology in polyps less than 10 mm does *not* require surveillance. Polyps less than 10 mm in size with villous histology are a rare event. Yet, more recent evidence does suggest that villous histology confers a slightly increased risk of CRC cancer incidence and mortality. However, this effect does appear to have less importance when polyp size is factored in.^19^

Wieszczy (2020) identified that individuals who had at least 1 adenoma with high-grade dysplasia of any size were at higher risk of developing CRC. However, number of adenomas or villous histology were not found to be independent risk factors for colorectal cancer incidence or mortality.^16^ Given the above available evidence, the panel determined that it was too premature to make a significant change regarding the surveillance of polyps with villous or tubulovillous histology. Thus, the follow-up recommendation is unchanged from 2013.

Voting results:

Decision achieved by consensus (9/10).

*Initial colonoscopy finding of:* More than 10 tubular adenoma(s).

**RECOMMENDATION:**

**For a colonoscopy finding of more than 10 tubular adenomas on a single colonoscopy, the panel recommends colonoscopy in 1 year and consider genetic counseling.**

Review of currently existing CPGs (Supplementary Appendix 2):

- ESGE recommends patients with 10 or more adenomas should be referred for genetic counselling.^5^
- USMSTF recommends patients with >10 adenomas completely removed at high-quality exam, repeat colonoscopy in 1 year.^6^
- Within Canada, CCO recommends that people with >10 adenomas undergo genetic assessment and receive a clearing colonoscopy within one year.^7^

Patients with >10 adenomas found on a single colonoscopy have an increased risk of hereditary polyposis syndromes (eg, familial adenomatous polyposis). Timely clearing colonoscopy is required to ensure that all adenomatous lesions have been removed. Multiple groups have recommended referral for genetic testing in all patients with >10 adenomas cumulatively over the patient’s lifetime.^23,24^ Given that Alberta has limited access to genetic testing, the panel suggested genetic counselling at the endoscopist’s discretion rather than an absolute recommendation.

Voting results:

Unanimous decision (10/10).

*Initial colonoscopy finding of:* 1 or 2 sessile serrated lesions <10 mm.

**RECOMMENDATION:**

**For a colonoscopy finding of 1 or 2 sessile serrated lesions <10 mm, the panel recommends colonoscopy in 5 years.**

Currently, there is significant variation used to describe serrated lesions in the colon including terms such as hyperplastic polyp, sessile serrated adenoma/polyp with or without dysplasia, and traditional serrated adenoma. The use of consistent nomenclature is required to improve communication and clarity of recommendations. The expert panel has adopted the term sessile serrated lesion (SSL) of the colon. This nomenclature is used by the Alberta Provincial GI Pathology Group (2020) and is consistent with established World Health Organization (WHO) 2019 terminology.^25–27^

Review of currently existing CPGs (Supplementary Appendix 2):

- ESGE recommends patients undergoing complete removal of any serrated polyp <10 mm without dysplasia do not require endoscopic surveillance and should be returned to screening.^5^ It should be noted that this guideline includes HPs within the category of SSL’s.
- USMSTF recommends colonoscopy in 5–10 years for 1–2 sessile serrated polyp(s) (<10 mm).^6^
- Within Canada, CCO recommends one or more sessile serrated adenoma(s) <10 mm without dysplasia should lead to a repeat colonoscopy in 5 years.^7^

Evidence for the development of metachronous CRC following removal of small (<10 mm) serrated lesions is still evolving. Two recent large retrospective cohort studies demonstrated non-significant hazard ratios compared to no polyps for small SSL’s removed from either the proximal or distal colon.^28^

In contrast, a large retrospective cohort study of 233 393 individuals identified that proximal small SSLs were associated with an increased risk of CRC with the risk beginning to rise after 3 years of follow-up (HR 2.6 [1.7–3.9]). There was no increased risk of CRC seen for small distal SSL’s.^29^

To better inform our decision, a literature review was performed. The research questions were:

Are patients with 1-2 SSLs at baseline colonoscopy at higher risk for CRC than:

- those with no polyps (normal colonoscopy)
- those with 1–2 TA’s
- the general population (never screened)

Full-text screening was completed by 1 reviewer from AHS Screening Programs with exclusions on the following criteria.

- If number of SSL ≤5 and CRC risk is not present.
- If not multicenter study.
- If N less than 1000.

As a result of the full-text screening only a single recently published meta-analysis was identified.^30^ See Supplementary Appendix 5. None of the studies included in the meta-analysis met our inclusion criteria. In the subgroup analysis between SSL’s alone and low-adenomas (LRA’s) alone, there was no difference between groups in metachronous Advanced colorectal neoplasia (ACRN) or CRC (OR 1.0; 95% CI, 0.18–5.52). In the analysis between SSL’s alone and High Risk Adenomas (HRA’s) alone, patients with SSL’s alone had a tendency to a lower risk of metachronous ACRN than those with HRAs alone however, this was not statistically significant (OR, 0.31; 95% CI, 0.07–1.44). It should be noted that in this meta-analysis, there was significant heterogeneity between studies with differing definitions used for HRA and ACRN.

This paucity of evidence is consistent with what has been reported by other jurisdictions around the world. Further research is needed regarding the impact of size, location, and number of SSLs on the development of metachronous colorectal cancer. In the absence of new evidence, the decision was to continue with the 2013 ACRCSP recommendation.

Voting results

Unanimous decision (10/10).

*Initial colonoscopy finding of:* 3–10 sessile serrated lesions <10 mm.

**RECOMMENDATION:**

**For a colonoscopy finding of 3–10 sessile serrated lesions <10 mm, the panel recommends colonoscopy in 3 years.**

Review of currently existing CPGs (Supplementary Appendix 2):

- ESGE recommends patients with complete removal of any serrated polyp <10 mm without dysplasia do not require endoscopic surveillance and should be returned to screening. The number of serrated lesions if <10 mm does not impact this decision.^5^
- USMSTF recommends colonoscopy in 3–5 years for 3–4 sessile serrated polyps (<10 mm) and colonoscopy in 3 years for 5–10 sessile serrated polyps (<10 mm).^6^
- Within Canada, CCO recommends one or more sessile serrated adenoma(s) <10 mm without dysplasia repeat colonoscopy in 5 years.^7^

The resolve this disparity, a literature review was performed. The research questions were:

1. Are patients with 3–4 SSLs at baseline at higher risk than:
  - those with no polyps
  - those with 1–2 SSLs
2. Are patients with 5–10 SSLs who do not meet the criteria for serrated polyposis syndrome at higher risk than those with 1–2 SSLs.

As a result of the full-text screening there were no studies that met the needs of our inclusion/exclusion criteria. This paucity of evidence is consistent with what has been reported by other jurisdictions around the world. Further research is needed regarding the impact of size, location, and number of SSLs on the development of metachronous colorectal cancer. The cut-off for this recommendation at 10 polyps is somewhat arbitrary. More than 10 sessile serrated lesions should raise the possibility of a serrated polyposis syndrome (see below). In the absence of new evidence, the decision was to continue with 2013 recommendation.

Voting results:

Unanimous decision (10/10).

*Initial colonoscopy finding of:* One or more sessile serrated lesion(s) >10 mm, or traditional serrated adenomas (any size) or SSL with dysplasia (any size).

**RECOMMENDATION:**

**For a colonoscopy finding of one or more sessile serrated lesion(s) >10 mm, or traditional serrated adenoma(s) (any size), or sessile serrated lesion with dysplasia (any size), the panel recommends colonoscopy in 3 years.**

There is consistent evidence that TSA, large SSL’s, and any SSL with dysplasia pose a significant increased risk for subsequent CRC.^11,29^ This is reflected in congruent recommendations from all expert groups. The recommendation for colonoscopy in 3 years is based on a similar risk for the development of CRC as for large adenomas. This recommendation is unchanged from 2013.

Voting results:

Unanimous decision (10/10).

*Initial colonoscopy finding of:* Serrated polyposis syndrome

**RECOMMENDATION:**

**For a colonoscopy finding of serrated polyposis syndrome (SPS), the panel recommends colonoscopy in 1 year.**

**Serrated polyposis syndrome is defined as: (1) at least 5 serrated lesions proximal to the rectum, with 2 or more that are >10 mm or; (2) more than 20 serrated lesions or polyps of any size distributed throughout the large bowel, with at least 5 proximal to the rectum.**

Serrated polyposis syndrome (SPS) is characterized by multiple serrated polyps found on colonoscopy and is associated with an increased risk of CRC.

The 2019 World Health Organization (WHO) guideline contains the following updated criteria for serrated polyposis syndrome (SPS) diagnosis:

1. ≥5 serrated lesions/polyps proximal to the rectum, all being ≥5 mm in size, with ≥2 being ≥10 mm in size, or;
2. More than 20 serrated lesions/polyps of any size distributed throughout the large bowel, with ≥5 being proximal to the rectum.

Any serrated polyp subtype (HP, SSL, and TSA) is to be included in the final polyp count and the polyp count is cumulative over multiple colonoscopies.^31^

Voting results:

Unanimous decision (10/10).

*Initial colonoscopy finding of:* Synchronous sessile serrated lesion and tubular adenoma.

**RECOMMENDATION:**

**For a colonoscopy finding of synchronous sessile serrated lesions and tubular adenomas, no recommendation made.**

Review of existing CPG’s:

- British Society of Gastroenterology (BSG) (2020) – “There is evidence to suggest that the future CRC risk may be additive between serrated and adenomatous polyps and their numbers should be summated when determining surveillance intervals.^3^”
- ESGE – “There is evidence that advanced adenoma with synchronous serrated polyp of any kind results in higher metachronous advanced neoplasia risk compared to advanced adenoma without synchronous serrated polyp. However, such patients would already be classified as in need of surveillance, regardless of the presence of serrated polyps. Any added value of combining adenomas with serrated polyp count to fulfill multiplicity criteria is therefore not supported.^5^”
- USMSTF – “Future research may clarify whether patients with a combination of <10mm SSPs and conventional adenomas have a distinct risk that should merit different management.^6^”
- CCO—No recommendation.

We identified 2 recent publications pertinent to this issue. One small study of 1389 patients^32^ compared those who had 1–2 TA’s (<10 mm) at baseline colonoscopy with patients who had 1–2 TA’s and 1–2 SSL’s (all <10 mm). The rate of total metachronous advanced adenoma in those with TA with SSL was also higher than the TA only cohort and approached significance (12.8% (5/39) versus 5.2% (59/113)); *P* = 0.056, respectively. A recent meta-analysis^29^ compared patients with SSL + LRA vs LRA alone. The odds ratio of subsequent ACRN was 1.5 (0.25–9.81). The panel determined that there was insufficient current evidence to make a recommendation for a colonoscopy finding of synchronous sessile serrated lesion and tubular adenoma.

Voting results:

Unanimous decision (10/10).

*Initial colonoscopy finding of:* Piecemeal resection of a large (≥10 mm) non-pedunculated polyp or lesion.

**RECOMMENDATION:**

**Following complete endoscopic piecemeal removal of a large (≥10 mm) non-pedunculated polyp or lesion, recommend first repeat endoscopic assessment** ^*^ **in 6 months.**

*For recto-sigmoid lesions, the choice of limited flexible sigmoidoscopy vs full colonoscopy is left to endoscopist’s discretion.

**Subsequent colonoscopy surveillance intervals** ^**^:

- **If the initial polyp was ≥20 mm, the next surveillance colonoscopy should be in 1 year. If no recurrence is detected at the resection site, the panel recommends subsequent colonoscopy surveillance in 3 years.**
- **If the initial polyp was ≥10 mm–19 mm, the next surveillance colonoscopy should be in 3 years** ^***^ **. If no recurrence is detected at the resection site, the panel recommends subsequent colonoscopy surveillance in 5 years.**

**Endoscopist discretion to perform surveillance at an earlier interval if concern for advance histological findings or other conflicting issues.

***Consideration for 12-month follow-up if high grade dysplasia, resection required multiple passes or challenging position noted.

Piecemeal resection is the resection of a ≥10mm non-pedunculated polyp or lesion, where more than one pass of the snare is required either due to size or polyp orientation.

Review of currently existing CPGs (Supplementary Appendix 2):

- ESGE “recommends a 3–6 month early repeat colonoscopy following piecemeal endoscopic resection of polyps ≥20 mm. A first surveillance colonoscopy 12 months after repeat colonoscopy is recommended to detect late recurrence.^5^”
- USMSTF recommends repeat colonoscopy (first surveillance) in 6 months for patients with piecemeal resection of adenoma or SSP >20 mm. Second surveillance 1 year from first surveillance, and third surveillance 3 years from the second surveillance.^6^
- Within Canada, CCO recommends a colonoscopy to check the polypectomy site within 6 months for large sessile polyp removed piecemeal. Subsequent surveillance recommendations are at the endoscopist’s discretion.^7^

A study of 1427 patients^33^ compared piecemeal vs. en-bloc polypectomy in 5–20 mm polyps. There was an increased risk of incomplete polyp resection in the piecemeal group (20% vs. 8.4%). Risk for incomplete resection was also associated with increased polyp size and histology (SSL’s > TA’s).

A systematic review of 38 studies^34^ identified that the risk of recurrence at *subsequent* scopes was: 20% (95% CI:16, 25) with piecemeal polypectomy vs 3% (95% CI: 2.5) in the en-bloc resection group. 75% of polyp recurrences were identified at 3 months and 96% at 6 months. Polyp size did not affect recurrence: 10–20 mm, 20–30 mm, and >30 mm polyps all had recurrence rates of 18–19%.

The panel identified that there is a lack of uniformity in the definition of piecemeal resection. For all lesions, it is key that a complete polypectomy with removal of all abnormal tissue is carried out. It is also recognized that polyp size is only one factor in determining risk of incomplete resection. Polyp location, orientation and morphology also play a role. The panel was also cognizant that there is a wide range in polypectomy ability among colonoscopists and any recommendation should reflect the skill level of the average endoscopist. Recommendations for subsequent polypectomy intervals after piecemeal resection are based on expert opinion only due to the current lack of evidence.

Voting results:

Unanimous decision (10/10).

*Initial colonoscopy finding of:* Subsequent colonoscopy surveillance after high-risk lesions.

**RECOMMENDATION:**

**High risk lesions require surveillance colonoscopy at 3 years and then subsequent colonoscopy in 5 years. If no polyps requiring surveillance are detected at both scopes, the panel recommends considering a return to average risk FIT screening.**

High risk lesions are defined as 5–10 tubular adenomas <10 mm, tubular adenoma ≥10 mm, tubular adenoma with villous or HGD, or 3–10 sessile serrated lesions <10 mm, any sessile serrated lesion ≥10 mm, any sessile serrated lesion with dysplasia or traditional serrated adenoma.

Review of currently existing CPGs (Supplementary Appendix 2):

- ESGE recommends a surveillance colonoscopy after 3 years for complete removal of at least 1 adenoma ≥10 mm or with high grade dysplasia, or ≥5 adenomas, or any serrated polyp ≥10 mm or with dysplasia. If that colonoscopy is normal, a colonoscopy in 5 years is recommended. If the 5-year colonoscopy is normal, the patient is to return to average-risk screening.^5^
- USMSTF recommends interval for next surveillance is based on findings at first surveillance. If normal colonoscopy or 1–2 small TA(s) then colonoscopy in 5 years. If 3–4 TA’s <10 mm colonoscopy in 3–5 years. If adenoma ≥10 mm or with tubulovillous/villous histology; or adenoma with high grade dysplasia; or 5–10 adenomas (<10 mm) then interval for next surveillance in 3 years. “New evidence is required to guide serial surveillance of individuals with SSPs and large HPs.^6^”
- Within Canada, CCO recommends subsequent colonoscopy based on the finding at 3 years. Subsequent surveillance was not addressed.^7^

The expert panel noted that these recommendations are supported by expert opinion only and thus recommendations should also reflect operational practicalities and clarity. As well, stopping rules may need to be enacted for patients who continue to receive surveillance colonoscopies for remote advanced lesions despite multiple normal subsequent colonoscopies.

Voting results:

Unanimous decision (10/10).

## Conclusions

In summary, our review underscores the balance between the benefits and potential drawbacks of surveillance colonoscopy following polypectomy in reducing the risk of subsequent colorectal cancer diagnosis. While this approach has demonstrated its efficacy when appropriately indicated, it is crucial to consider the inherent risks, patient discomfort, cost, and inconvenience associated with colonoscopy.

Colonoscopy complications include severe bleeding and perforation, emphasizing the need to minimize the burden and risk of follow-up colonoscopies whenever possible. Complication risk may vary according to colonoscopy indication. For instance, a comprehensive review of complications in screening colonoscopies found that the rate of severe bleeding ranged from 16.4 to 36.18 per 10 000 colonoscopies, while the rate of perforation ranged from 7.62 to 8.50 per 10 000 colonoscopies.^35^ However, a recent Canadian study in FIT positive patient undergoing colonoscopy identified significant bleeding in 26 (95% CI 22–30) per 10 000 colonoscopies and death in 3 (95% CI 1–10) per 100 000 colonoscopies.^36^

It is crucial to acknowledge specific limitations in our work. One significant constraint revolves around the scarcity of robust evidence, particularly concerning the long-term risk of developing colorectal cancer after the removal of sessile serrated lesions (SSLs). Notably, the limited number of available studies, some of which encompassed both hyperplastic polyps and SSLs, may have included a substantial proportion of patients at a lower risk for developing colorectal cancer. Consequently, our recommendations for the surveillance of these lesions are largely unchanged from the 2013 guideline. As well, there was very little evidence for post-polypectomy outcomes in those 45–50 years old age. Consequently, this group was outside the scope of this project.

Another limitation concerns the utility of the Agree II tool in transparently selecting appropriate clinical practice guidelines (CPGs). While the Agree II tool is widely recognized for assessing the rigor and transparency of CPG development, we observed instances where Agree II scores did not align with clinical relevance, particularly in the context of guidelines from other regions. For instance, certain guidelines from the United Kingdom and Australia had high Agree II scores but lacked applicability in the Alberta context due to differences in lesion categorization and recommendations on surveillance intervals. For example, UK recommendations assume the existence of a population based screening program with invitation letters—a situation not applicable to Alberta. We also acknowledge that Alberta is an outlier regarding the FIT screening interval, being the only province to have a frequency of 1–2 years. This potentially makes the follow-up recommendations regarding FIT less relevant to other Canadian provinces. The Canadian Task Force on Preventive Health suggests a screening interval of every 2 years for FIT.

Additionally, we acknowledge the potential for bias among our panel members, some of whom are closely affiliated with the provincial screening program. However, it is worth noting that these members bring valuable expertise and practical insights into the subject matter and the challenges of program implementation in Alberta.

In conclusion, our review aligns with emerging evidence suggesting less intense surveillance for tubular adenomas. However, due to the uncertainty surrounding the surveillance of sessile serrated lesions, our recommendations in this regard have remained unchanged. As the field of colorectal cancer surveillance continues to evolve, ongoing research and collaboration will be essential to refine and enhance our recommendations for optimizing patient outcomes while minimizing the burdens associated with colonoscopy surveillance.

The ACRCSP would like to thank the following external reviewers for their valuable input into this document:

- Dr. Jennifer Telford, Clinical Professor of Medicine, University of British Columbia
- Dr. Alan Barkun, Professor of Medicine, McGill University
- Dr. Harminder Singh, University of Manitoba
- Dr. Jerry McGrath, Memorial University
- Dr. Catherine Dubé, University of Ottawa.
- Dr. Ross Stimpson, University of Manitoba.
- Dr. Sander Velduyzen van Zanten, University of Alberta

## Supplementary Material

gwae007_suppl_Supplementary_COI_Statement

gwae007_suppl_Supplementary_Material

## Data Availability

The data underlying this article are available in the article and in its online supplementary material.
