# Supplementary material for: Post-polypectomy surveillance: follow-up recommendations from the Alberta Colorectal Cancer Screening Program
Source: J Can Assoc Gastroenterol. 2024 Mar 19;7(4):319–28. doi: 10.1093/jcag/gwae007 (PMC11317626; doi:10.1093/jcag/gwae007)
Supplement: gwae007_suppl_Supplementary_Material [file gwae007_suppl_supplementary_material.docx]

### APPENDIX 1: Post-Polypectomy Colonoscopy Surveillance CPG AGREE II Scores

|  |  | Average score between 2 appraisers - Scores are between 1 to 7, using AGREE II | | | | |
| --- | --- | --- | --- | --- | --- | --- |
|  |  | **United Kingdom,** (Rutter, *et al.* 2019) | **Europe,** (Hassan, *et al.* 2020) | **Ontario,** (Dubé, *et al.* 2019) | **Australia,** (Barclay, *et al.* 2019) | **United States,** (Gupta, *et al.* 2020) |
| **Scope & Purpose** | 1. Objectives | 7 | 6.5 | 7 | 7 | 7 |
|  | 1. Health question | 7 | 6 | 2.5 | 7 | 7 |
|  | 1. Target population | 7 | 5.5 | 5.5 | 7 | 7 |
| Domain Score |  | 100% | 83% | 67% | 100% | 100% |
| **Stakeholder Involvement** | 1. Relevant professional groups represented | 5.5 | 2.5 | 6.5 | 7 | 5 |
|  | 1. Target population preferences | 6 | 6 | 1 | 7 | 2.5 |
|  | 1. Target users defined | 4.5 | 6.5 | 5.5 | 7 | 1 |
| Domain Score |  | 72% | 67% | 56 % | 100% | 31% |
| **Rigor of Development** | 1. Systematic search conducted | 7 | 3 | 5.5 | 7 | 7 |
|  | 1. Selection criteria described | 7 | 2 | 5.5 | 7 | 7 |
|  | 1. Evidence strengths and limitations described | 7 | 7 | 5.5 | 7 | 7 |
|  | 1. Methods used to formulate recommendations described | 7 | 6.5 | 3.5 | 7 | 5 |
|  | 1. Benefits, side effects, risks considered | 7 | 6.5 | 7 | 7 | 6 |
|  | 1. Link between recommendations and evidence | 7 | 7 | 6.5 | 5.5 | 7 |
|  | 1. External review by experts | 2.5 | 5.5 | 6 | 6 | 1 |
|  | 1. Updating procedure described | 6 | 6 | 3 | 7 | 1 |
| Domain Score |  | 89% | 74% | 72% | 95% | 69 % |
| **Clarity of Presentation** | 1. Specific, unambiguous recommendations | 7 | 7 | 7 | 7 | 7 |
|  | 1. Different management options presented | 7 | 7 | 6 | 7 | 6.5 |
|  | 1. Key recommendations easily identifiable | 7 | 7 | 7 | 7 | 7 |
| Domain Score |  | 100% | 100% | 94% | 100% | 97% |
| **Applicability** | 1. Facilitators and barriers discussed | 7 | 6 | 1 | 6 | 4.5 |
|  | 1. Support materials provided | 7 | 6 | 6 | 7 | 5.5 |
|  | 1. Resource implications considered | 7 | 6.5 | 1 | 7 | 1 |
|  | 1. Monitoring or audit criteria presented | 6 | 6 | 3 | 1 | 1 |
| Domain Score |  | 96% | 85% | 29% | 71% | 33% |
| **Editorial Independence** | 1. Editorially independent from funding body | 5 | 1 | 1 | 6.5 | 6.5 |
|  | 1. Competing interests reported | 6.6 | 6 | 2 | 7 | 6 |
| Domain Score |  | 79% | 42% | 8% | 96% | 88% |
| **Overall Assessment** | | **92%** | **75%** | **58%** | **92%** | **67%** |

### APPENDIX 2: Initial Colonoscopy Findings of Normal or Hyperplastic Polyp(s), Tubular Adenoma(s), and Sessile Serrated Lesion(s)

| Colonoscopy  Findings: | **Europe**  **ESGE**  (Hassan, et al. 2020) | **United States**  **USMSTF**  (Gupta, et al. 2020) | **Ontario**  **CCO**  (Dubé, et al. 2019) | **Alberta**  **ACRCSP 2023 (new)** | **Alberta**  **ACRCSP 2013** |
| --- | --- | --- | --- | --- | --- |
| No polyps | Return to screening | Colonoscopy in  10 years | FIT in 10 years | No colonoscopic surveillance, FIT in 10 years | FIT in 10 years |
| Hyperplastic polyp(s)  <10mm | No  recommendation^[[1]](#endnote-1)^ | Colonoscopy in 10 years (or other screening modality) if  ≤20 in rectum or  sigmoid colon or;  Colonoscopy in 10 years if ≤20 proximal to  sigmoid colon | FIT in 10 years (HP in rectum or sigmoid) | FIT in 10 years^[[2]](#endnote-2)^ | Maintain screening interval based on underlying risk level (consider as normal) |
| Hyperplastic polyp(s)  ≥10mm | 3 years^[[3]](#endnote-3)^ | Colonoscopy in 3-5 years for HP ≥10mm^[[4]](#endnote-4)^ | No recommendation | Colonoscopy in 3 years  if HP ≥10mm proximal to sigmoid colon^[[5]](#endnote-5)^  Colonoscopy in 5 years if HP ≥10mm in rectosigmoid | Colonoscopy in 5 years, if ≥4 HP proximal to sigmoid or any HP >5mm proximal to sigmoid |
| 1-2 Tubular Adenoma(s) <10mm | Return to screening program  (or colonoscopy in 10 years if no screening program exists) | Colonoscopy  7- 10 years | FIT in 5 years | FIT in 5 years | Colonoscopy  5 – 10 years |
| 3-4 Tubular Adenomas <10mm |  | 3-5 years | 3 years | Colonoscopy in  5 years | 3 years |
| 5-10 Tubular Adenomas <10mm | 3 years | 3 years |  | 3 years |  |
| ≥10mm in size |  |  |  |  |  |
| High Grade Dysplasia |  |  |  |  |  |
| Colonoscopy  Findings: | **Europe**  **ESGE**  (Hassan, et al. 2020) | **United States**  **USMSTF**  (Gupta, et al. 2020) | **Ontario**  **CCO**  (Dubé, et al. 2019) | **Alberta**  **ACRCSP 2023 (new)** | **Alberta**  **ACRCSP 2013** |
| Villous/Tubulovillous | Return to screening program^[[6]](#endnote-6)^ | 3 years | 3 years | 3 years | 3 years |
| >10 Tubular Adenomas | Genetic counselling | 1 year and genetic counselling | Within 1 year and genetic assessment^[[7]](#endnote-7)^ | Within 1 year and genetic counselling^[[8]](#endnote-8)^ | <3 years |
| Large adenoma piecemealed | Colonoscopy 3-6 months following piecemeal of polyps ≥20mm | Colonoscopy in 6 months following piecemeal of adenoma ≥20mm | N/A | Colonoscopy in 6 months following piecemeal of adenoma ≥10mm | N/A |
| 1-2 SSP <10 mm in size | Any serrated polyp without dysplasia  <10 mm: Return to screening program  (or colonoscopy in  10 years if no screening program exists). | Colonoscopy in  5-10 years | Colonoscopy in  5 years | Colonoscopy in  5 years | Colonoscopy in  5 years |
| 3-4 SSP <10 mm in size |  | Colonoscopy in  3-5 years |  | Colonoscopy in  3 years | Colonoscopy in  3 years |
| 5-10 SSP <10 mm in size |  | Colonoscopy in  3 years |  |  |  |
| ≥10mm in size  (any number) | Colonoscopy in  3 years |  | Colonoscopy in  3 years |  |  |
| [with] dysplasia  (any size) |  |  |  |  |  |
| Traditional serrated adenoma (any size) |  |  |  |  | Colonoscopy in:  3 years if dysplasia;  5 years if 1-2 <10mm or no dysplasia |
| [large] SSP removed piecemeal | Colonoscopy in  3-6 months following piecemeal of polyps >20mm | Colonoscopy in  6 months | Colonoscopy in  ≤6 months | Colonoscopy in  ≤6 months | Repeat colonoscopy in  2-6 months, then  3 years |
| Serrated polyposis syndrome | No recommendation^[[9]](#endnote-9)^ | No recommendation^[[10]](#endnote-10)^ | Colonoscopy in  1 year^[[11]](#endnote-11)^ | Colonoscopy in  1 year^[[12]](#endnote-12)^ | Colonoscopy in  1 year |

ESGE: European Society of Gastrointestinal Endoscopy; USMSTF: United States Multi-Society Task Force; CCO: Cancer Care Ontario; ACRCSP: Alberta Colorectal Cancer Screening Pr

### APPENDIX 3: Summary of 2023 recommendations from guideline committee

| **Initial colonoscopy findings** | | | **Recommendation** | | **Level of agreement**  Agree Disagree | |
| --- | --- | --- | --- | --- | --- | --- |
| 1. | Normal or no polyps | | For an average risk patient with no polyps or normal findings on colonoscopy, recommend FIT in 10 years. | | 100% (consensus reached) | |
| 2. | Hyperplastic polyp(s) <10mm | | For an average risk patient with finding(s) of hyperplastic polyp(s) <10mm, recommend FIT in 10 years^*^.  ^*More than 20 hyperplastic polyps, especially if found proximal to the sigmoid colon, should lead to consideration of serrated polyposis syndrome.^ | | 100% (consensus reached) | |
| 3. | Hyperplastic polyp(s) ≥10mm | | For a colonoscopy finding of hyperplastic polyp(s) ≥10mm:   1. Proximal to sigmoid colon, recommend colonoscopy in 3 years^*^. 2. In rectosigmoid, recommend colonoscopy in 5 years.   ^*Hyperplastic polyp(s) proximal to sigmoid colon should be considered sessile serrated lesion (SSL) with colonoscopy surveillance in 3 years.^ | | 100% (consensus reached) | |
| 4. | 1 or 2 tubular adenoma(s) <10mm | | For a colonoscopy finding of 1 or 2 tubular adenoma(s) <10mm, recommend FIT in 5 years. | | 100% (consensus reached) | |
| 5. | 3 or 4 tubular adenomas <10mm | | For a colonoscopy finding of 3 or 4 tubular adenomas <10mm, recommend colonoscopy in 5 years. | | 100% (consensus reached) | |
| 6. | | 5 to 10 tubular adenomas <10mm, or  any adenoma ≥10mm, or with villous/tubulovillous features or high-grade dysplasia | | For a colonoscopy finding of 5 to 10 tubular adenomas <10mm, or any adenoma ≥10mm, or with villous/tubulovillous features or high-grade dysplasia, recommend colonoscopy in 3 years. | | 90% (consensus reached)  One member disagreed with the 3-year recommendation, citing that the finding of high-grade dysplasia may warrant an earlier follow-up depending on polyp morphology and size. |
| 7. | | >10 tubular adenoma(s) | | For a colonoscopy finding of more than 10 tubular adenomas, recommend colonoscopy in 1 year and genetic counselling^*^.  ^*Consider genetic testing referral. Patients with >10 adenomas found on colonoscopy have an increased risk for hereditary polyposis. Timely clearing colonoscopy is required to ensure that all adenomatous lesions have been removed.^ | | 100% (consensus reached) |
| 8. | | 1 or 2 sessile serrated lesions <10mm | | For a colonoscopy finding of 1 or 2 sessile serrated lesions <10mm, recommend colonoscopy in 5 years. | | 100% (consensus reached) |
| 9. | | 3 to 10 sessile serrated lesions <10mm | | For a colonoscopy finding of 3 to 10 sessile serrated lesions <10mm, recommend colonoscopy in 3 years. | | 100% (consensus reached) |
| 10. | | One or more sessile serrated lesion(s) >10mm, or traditional serrated adenomas (any size) or SSL with dysplasia (any size) | | For a colonoscopy finding of one or more sessile serrated lesion(s) >10mm, or traditional serrated adenoma(s) (any size), or sessile serrated lesion with dysplasia (any size), recommend colonoscopy in 3 years. | | 100% (consensus reached) |
| 11. | | Serrated polyposis syndrome | | For a colonoscopy finding of serrated polyposis syndrome (SPS), recommend colonoscopy in 1 year.  Serrated polyposis syndrome: *1) at least five serrated lesions proximal to the rectum, with two or more that are >10mm or; 2) more than 20 serrated lesions or polyps of any size distributed throughout the large bowel, with at least five proximal to the rectum.* | | 100% (consensus reached) |
| 12. | | Synchronous sessile serrated lesion and tubular adenoma | | For a colonoscopy finding of synchronous sessile serrated lesion and tubular adenoma, no recommendation made. | | 90% (consensus reached) |
| 13. | | Piecemeal resection of a large (≥10mm) non-pedunculated polyp or lesion | | Following complete endoscopic piecemeal^*^ removal of a large (≥10mm) non-pedunculated polyp or lesion, recommend first repeat endoscopic assessment in 6 months^**^.  ^*^*^Piecemeal resection is the resection of a ≥10mm non-pedunculated polyp or lesion, where more than one pass of the snare is required either due to size or polyp orientation.^*  ^**For recto-sigmoid lesions, choice of limited flexible sigmoidoscopy vs full colonoscopy is at endoscopist’s discretion.^  Subsequent colonoscopy surveillance intervals^***^:   - If polyp ≥20mm, next surveillance colonoscopy in 1 year. If no reoccurrence detected at site, recommend subsequent surveillance in 3 years. - If polyp ≥10mm-19mm, next surveillance colonoscopy in 3 years^****^. If no reoccurrence detected at site, recommend subsequent surveillance in 5 years.   ^***Endoscopist discretion to perform surveillance at an earlier interval if concern for advance histological findings or other conflicting issues.^  ^****Consideration for 12-month follow-up if high grade dysplasia, resection required multiple passes or challenging position noted^*.* | | 100% (consensus reached) |
| 14. | | Subsequent colonoscopy surveillance after high-risk lesions | | High risk lesions^*^ require surveillance colonoscopy at 3 and then 5 years. If no polyps requiring surveillance are detected at both scopes, consider return to average risk FIT screening.  ^*High risk lesions: tubular adenomas 5-10 (<10mm), ≥10mm, villous or HGD, or sessile serrated lesions 3-10 (<10mm), ≥10mm, TSA and HGD.^ | | 100% (consensus reached) |

### APPENDIX 4: PRISMA 2020 flow diagram for evidence review of # of adenomas and risk of CRC

Reports excluded:

Reason 1: # of adenoma not discussed (n = 17)

Reason 2: sample less than 1000 (n =1 )

Reason 3: duplicate data set (n= 1)

Reason 4: # of CRC events not discussed (1)

Reports not retrieved

(n = 0)

Records excluded using multicentre/center filter

(n = 318)

Studies included in review

(n = 5)

Reports assessed for eligibility

(n = 25)

Reports sought for retrieval

(n = 25)

Records screened

(n = 343)

Records removed *before screening*:

Duplicate records removed (n = 139)

Records marked as ineligible by automation tools (n = 0)

Records removed for other reasons (n = 0)

Records identified from:

Databases (n = 482)

Registers (n = 0)

**Screening**

**Included**

**Identification**

**Identification of studies via databases and registers**

*From:* Page MJ, McKenzie JE, Bossuyt PM, Boutron I, Hoffmann TC, Mulrow CD, et al. The PRISMA 2020 statement: an updated guideline for reporting systematic reviews. BMJ 2021;372:n71. doi: 10.1136/bmj.n71.

For more information, visit: <http://www.prisma-statement.org/>

### APPENDIX 5: PRISMA 2020 flow diagram for evidence review of # of sessile serrated polyps and risk of CRC

**Identification of studies via databases and registers**

Records removed *before screening*:

Duplicate records removed (n = 586)

Records identified from *MEDLINE (Ovid); EMBASE; PubMed; Web of Science*

Databases (n = 767)

**Identification**

Records screened manually for multicentre studies

(n = 181)

Records excluded using multicentre study criteria

(n = 160)

Reports screened at abstract

(n = 21)

Reports excluded at abstract

(n = 4)

**Screening**

Reports excluded:

Reason 1: # of SSA not discussed (n = 2)

Reason 2: full study not published (n= 2)

Reports assessed for eligibility

(n = 4)

Studies included in review

(n = 1)

**Included**

*From:* Page MJ, McKenzie JE, Bossuyt PM, Boutron I, Hoffmann TC, Mulrow CD, et al. The PRISMA 2020 statement an updated guideline for reporting systematic reviews. BMJ 2021;372:n71. doi: 10.1136/bmj.n71.

### For more information, visit: <http://www.prisma-statement.org/>

APPENDIX 6: Literature Review Number of Adenomas and Subsequent Risk of Colorectal cancer

To assess the impact of the quantity of adenomas on the subsequent risk of developing colorectal cancer, a comprehensive literature search was conducted by two independent medical librarians affiliated with AHS Screening Programs. The search encompassed peer-reviewed databases such as MEDLINE, PubMed, PubMed Central, CINAHL, MEDLINE (Ebsco), and Web of Science, as well as grey literature sources like AHS Insite, OAISter, ASCO, ESMO Google, and Google Scholar. The inclusion criteria were limited to publications dated from July 16, 2010, to July 16, 2021.

During the full-text screening process, publications were excluded based on the following criteria:

- If the study did not involve individuals with ≤5 adenomas and did not assess colorectal cancer risk.
- If the study was not conducted at multiple centers.
- If the sample size (N) was less than 1000 participants.

Studies that investigated the relationship between the number of adenomas and the subsequent risk of colorectal cancer were included in the analysis if they met the following criteria: multi-center randomized controlled studies, cohort studies, or systematic reviews with a participant count exceeding 1000 colonoscopies.

Following the removal of duplicate publications, a total of 343 screened articles underwent full-text assessment by two independent reviewers serving on the scientific committee. This process resulted in the inclusion of 5 studies for our analysis. The PRISMA 2020 Prisma flow diagram is found in Appendix 4.

**Table A1. Brief Description of Included Studies for number of adenoma and CRC incidence**

| **Anderson, JC et al. 2018** | Can the Sum of Adenoma Diameters (Adenoma Bulk) on Index Examination Predict Risk of Metachronous Advanced Neoplasia? |
| --- | --- |
| **Population** | N=1948 patients, 62.7% were men, mean age was 58.0 years (SD, 6.7) |
| **Timeframe** | Mean of 45.2 months (3.75 years) follow-up (range, 22.6 to 97.2 mo). |
| **Outcome** | Adenoma bulk, the sum of diameters of all baseline adenomas, regardless of advanced features. |
| **Study** | Data were collected prospectively in a multicenter adenoma- chemoprevention trial (2004 to 2013). 11 centers in the USA. |

| **Atkin, W, Wooldrage, K et al. 2017** | Adenoma surveillance and colorectal cancer incidence: a retrospective, multicentre, cohort study |
| --- | --- |
| **Population** | N=11 944 intermediate-risk patients, 55% were men, median age was 66·7 years (IQR 58·4–74·0) |
| **Timeframe** | Median of 7.9 years follow-up. |
| **Outcome** | Heterogeneity in colorectal cancer incidence in intermediate-risk patients and the effect of surveillance on colorectal cancer incidence. |
| **Study** | Retrospective, multicentre, cohort study using routine lower gastrointestinal endoscopy and pathology data from patients who, after baseline colonoscopy and polypectomy, were diagnosed with intermediate- risk adenomas mostly (>99%) between Jan 1, 1990, and Dec 31, 2010, at 17 hospitals in the UK. |

| **Click, B et al. 2018** | Association of colonoscopy adenoma findings with long-term colorectal cancer incidence |
| --- | --- |
| **Population** | N=15 935 patients, 59.7% were men, median age was 64 years (IQR, 61-68) |
| **Timeframe** | Median of 13 years follow-up. |
| **Outcome** | CRC incidence within 15 years of the baseline colonoscopy. The secondary outcome was CRC mortality. |
| **Study** | Multicenter, prospective cohort study of participants in the Prostate, Lung, Colorectal, and Ovarian (PLCO) Cancer randomized clinical trial of flexible sigmoidoscopy (FSG) beginning in 1993 with follow-up for CRC incidence to 2013 across the United States. |

| **Cross, AJ et al. 2020** | Long-term colorectal cancer incidence after adenoma removal and the effects of surveillance on incidence: a multicentre, retrospective, cohort study |
| --- | --- |
| **Population** | N=11 852 intermediate- risk patients, 44% were women, median age was 66 years (IQR 58 to 74) |
| **Timeframe** | Median of 9.1 years follow-up for intermediate risk. |
| **Outcome** | CRC incidence and effects of surveillance on incidence among each risk group (low- risk, intermediate- risk and high- risk). |
| **Study** | Retrospective study 17 UK hospitals, mostly from 2000 to 2010. Patients were followed up through 2016. |

| **Wieszczy, P et al. 2020** | Colorectal Cancer Incidence and Mortality After Removal of Adenomas During Screening Colonoscopies |
| --- | --- |
| **Population** | N=236,089 patients, 37.8% were men, median age was 56 years |
| **Timeframe** | Median of 7.1 years follow-up. |
| **Outcome** | Colorectal cancer incidence and colorectal cancer death. |
| **Study** | Population-based cohort study. 132 centers in the Polish National Colorectal Cancer Screening Program, from 2000 through 2011. |

**Table A2. Evidence Table from Included Studies for number of adenoma and CRC incidence**

| Study | 1-2 TA | | No adenoma | | Risk | ≥3 TA | | No adenoma | | Risk |
| --- | --- | --- | --- | --- | --- | --- | --- | --- | --- | --- |
|  | Events | Total | Events | Total |  | Events | Total | Events | Total |  |
| 1. Anderson, JC et al., 2018 | 114 | 1410 |  |  |  | 22 | 164 |  |  |  |
| 1. Atkin, W, Wooldrage, K et al., 2017 | 200 | 10,915 |  |  |  | 10 | 1029 |  |  |  |
| 1. Click, B et al., 2018 | 48  *(1-2TA)* | 4496 | 71 | 7985 | ARR  1.2 (0.5 to 2.9)  *P*=0.19 | 7  *(≥3 TA)* | 572 | 71 | 7985 | ARR  1.3 (0.9 to 1.9)  *P*=0.73 |
| 1. Cross, AJ et al., 2020 | 195 *(Low risk)* | 14,401 |  |  |  | 14 *(intermediate risk)* | 1006 |  |  |  |
| 1. Wieszczy, P et al., 2020 | 58 *(Low risk,*  *1-2TA)* | 26,536 | 309 | 194,311 | SIR  0.35 (0.26 to 0.45) | 72 *(High risk, ≥3 TA)* | 15,242 | 309 | 194,311 | SIR  0.65 (0.51 to 0.82) |

For our clinical scenarios, we separated our analysis into 3 comparisons: >3 adenomas vs < 3 adenomas, 1-2 tubular adenomas versus 0 adenomas and > 3 adenomas vs 0 adenomas.

**Analysis 1: ≥3 vs <3 adenomas**

Figure A1 shows the group comparison between number of adenomas and the risk of CRC events (including advanced neoplasia). No significant difference in risk of CRC was identified between the patients with ≥3 or <3 adenomas. However, there was considerable heterogeneity between studies which may be the result of variable lengths of follow-up and differing patient related outcomes.


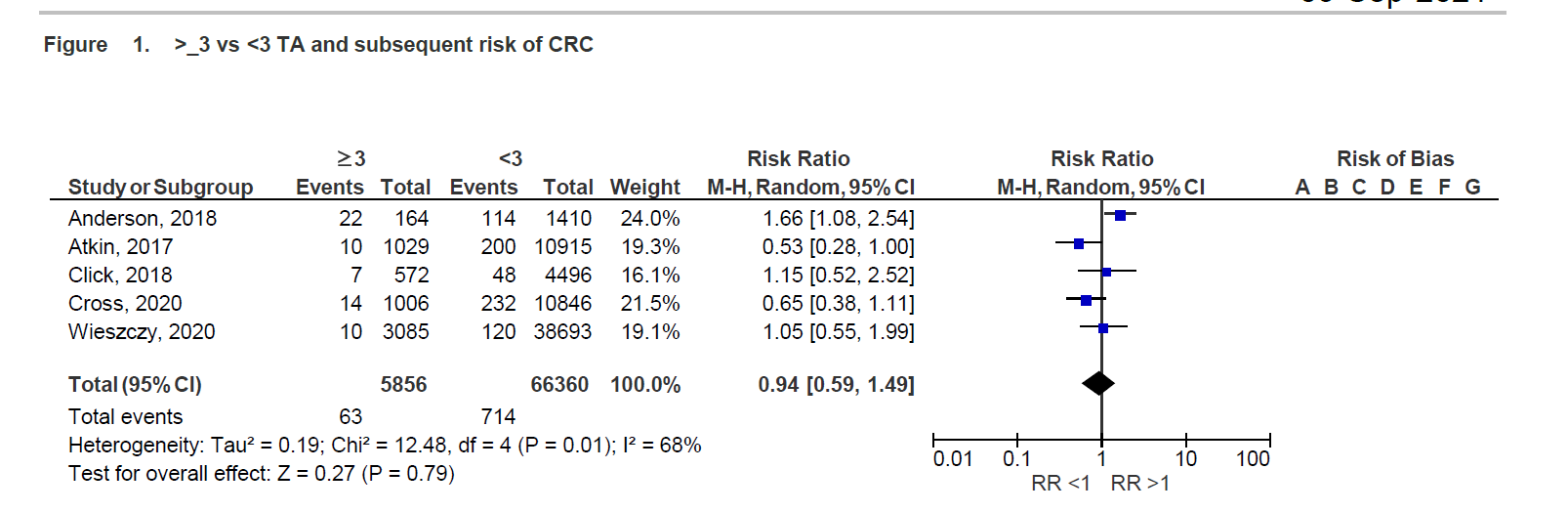
**Figure A1**: **≥ 3 vs. < 3 TA and subsequent risk of CRC**

**Analysis 2: 1-2 TA’s vs 0 TA’s**

Using the studies from the previous meta-analysis, a revised analysis was done comparing the risk ratio for 1-2 TA vs absence of adenoma. Out of the five studies, only Click (2018) and Wieszczy (2020) provided sufficient data to do a comparison. Overall, the risk ratio of 1.31 (95% CI 1.05, 1.63) indicates a slightly increased risk of CRC incidence with the presence of 1-2 TA compared to those with no TA’s (Figure A2). This evidence is limited by the fact that neither polyp size nor pathology was evaluated in determining the subsequent risk of colorectal cancer.

**Figure A2: 1-2 TA vs 0 TA and subsequent risk of CRC**


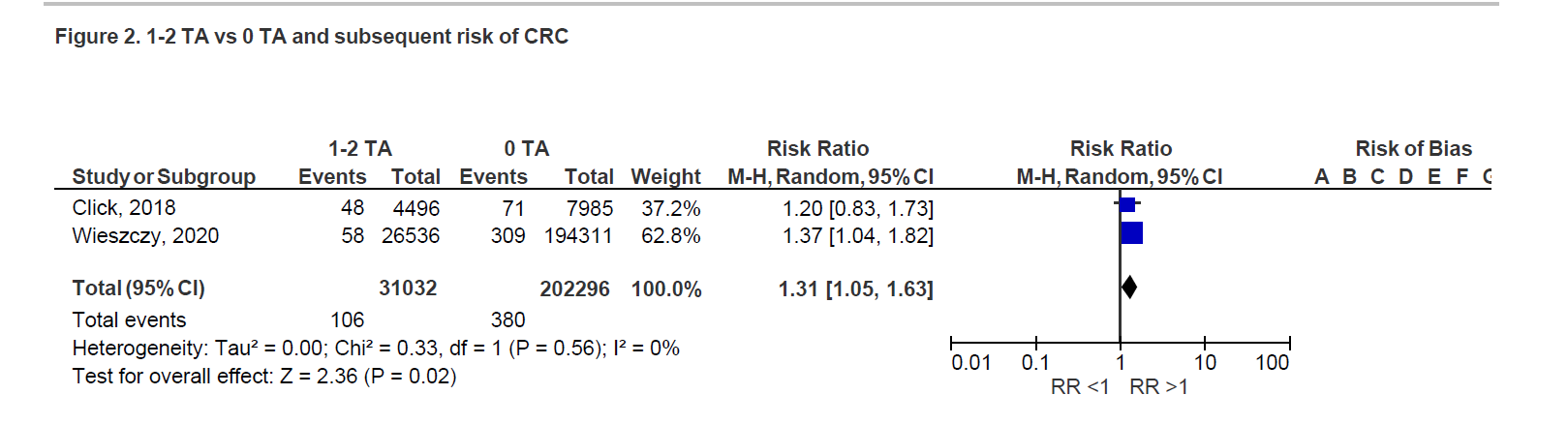


**Analysis 3: 3-4 TA’s vs vs 0 TA’s**

Using the studies from the previous meta-analysis, a revised analysis comparing the risk ratio for 3-4 TA vs no adenomas was performed. Out of the five studies included for review, only Click (2018) and Wieszczy (2020) had a comparison addressing the number of adenomas, however the analysis did not account for polyp size or advanced histology. When comparing patients with ≥ 3 adenomas to patients with 0 adenomas, there does appear to be an increased risk of subsequent CRC: RR: 2.21 (95% CI; 1.06- 4.62).

**Figure A3: ≥ 3 TA vs 0 TA and subsequent risk of CRC**

**
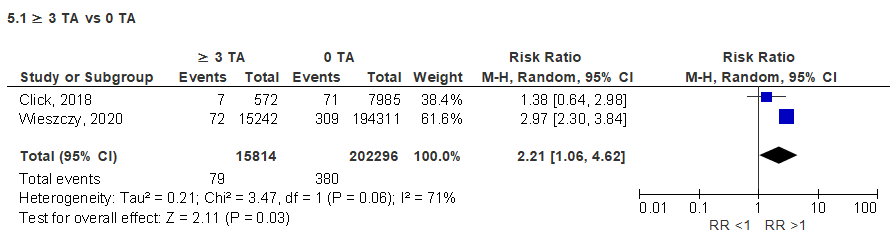
**

1. ESGE recommends that any serrated polyp <10 mm without dysplasia does not require endoscopic surveillance and should return to screening. If organized screening not available, repetition of colonoscopy 10 years after index procedure recommended. [↑](#endnote-ref-1)
2. More than 20 HP’s, especially if found proximal to the sigmoid colon, should lead to consideration of serrated polyposis syndrome. [↑](#endnote-ref-2)
3. Serrated polyp ≥10mm and with dysplasia yield similar metachronous advanced neoplasia or CRC and require surveillance at 3 years. [↑](#endnote-ref-3)
4. A 3-year follow-up is favored if concern about consistency in distinction between sessile serrated polyp and hyperplastic locally, bowel prep or complete excision, whereas a 5-year interval is favored if low concerns for consistency in distinction, adequate bowel prep and confident complete excision. [↑](#endnote-ref-4)
5. HP ≥10mm proximal to sigmoid colon should be considered sessile serrated lesion (SSL) and colonoscopy surveillance in 3 years. [↑](#endnote-ref-5)
6. Return to screening program or colonoscopy in 10 years if no screening program exists. [↑](#endnote-ref-6)
7. People with >10 adenomas should undergo genetic assessment for familial adenomatous polyposis syndromes. The subsequent surveillance interval will depend on the results of the genetic assessment and whether the colonoscopy is cleared of polyps. [↑](#endnote-ref-7)
8. Consideration for genetic testing referral. Patients with >10 adenomas found on colonoscopy have an increased risk for hereditary polyposis. Timely clearing colonoscopy is required to ensure that all adenomatous lesions have been removed. [↑](#endnote-ref-8)
9. High risk conditions, such as those with serrated polyposis syndrome or hereditary syndromes should receive an individualized surveillance schedule. [↑](#endnote-ref-9)
10. Patients with cumulative >20 hyperplastic polyps distributed throughout the colon, with at least five being proximal to the rectum, as well as those with five serrated polyps proximal to the rectum >5mm, with at least two ≥10mm meet criteria for serrated polyposis syndrome and may require specialized management. [↑](#endnote-ref-10)
11. Serrated polyposis syndrome: At least five serrated polyps proximal to the sigmoid colon, two of which are greater than 10mm; or any number of serrated occurring proximal to the sigmoid colon in someone who has a first degree relative with serrated polyposis; or more than 20 serrated polyps of any size throughout the colon. [↑](#endnote-ref-11)
12. Serrated polyposis syndrome: At least five serrated lesions proximal to the rectum, with two or more that are >10mm, or more than 20 serrated lesion or polyps of any size distributed throughout the large bowel, with at least five proximal to the rectum. [↑](#endnote-ref-12)
